# Current norms and practices in using a seizure diary for managing epilepsy: A scoping review

**DOI:** 10.4102/safp.v64i1.5540

**Published:** 2022-09-22

**Authors:** Chika K. Egenasi, Anandan A. Moodley, Wilhelm J. Steinberg, Anthonio O. Adefuye

**Affiliations:** 1 Department of Family Medicine, Faculty of Health Sciences, University of the Free State, Bloemfontein, South Africa; 2 Department of Neurology, Faculty of Health Sciences, University of KwaZulu-Natal, Durban, South Africa; 3 Division of Health Sciences Education, Faculty of Health Science, University of the Free State, Bloemfontein, South Africa

**Keywords:** seizure diary, epilepsy, paper-based seizure diary, electronic diary, seizure frequency, scoping review, articles, literature

## Abstract

**Background:**

Epilepsy is a chronic and debilitating condition affecting people of all ages in many nations. Healthcare practitioners look for effective ways to track patients’ seizures, and a seizure diary is one of the methods used. This scoping review sought to identify current norms and practices for using seizure diaries to manage epilepsy.

**Method:**

A scoping review was performed by screening relevant studies and identifying themes, categories and subcategories.

**Results:**

A total of 1125 articles were identified from the database; 46 full-text articles were assessed for eligibility, of which 23 articles were selected. The majority (48%) of the studies were prospective studies. The majority (65%) of the articles were studies conducted in the United States. The themes identified were types of seizure diaries used in clinical practice, contents and structure of a standardised seizure diary, the use and efficacy of seizure diaries in medicine and challenges relating to using a seizure diary for patient management.

**Conclusion:**

The study revealed that a seizure diary remains a relevant tool in managing epilepsy. The two forms of diaries in use are electronic and paper-based diaries. The high cost of data and the expensive devices required to access electronic diaries make it unsuitable in a resource-limited setting. Despite its disadvantages, imperfections and inadequacies, the paper-based diary is still relevant for managing patients with epilepsy in resource-limited settings.

**Contribution:**

This study reviewed the literature to find the current norms and practices in using seizure diaries. The benefits of the different formats were emphasised.

## Introduction

Epilepsy is a common chronic medical condition which is associated with physical risk and psychological and socio-economic consequences that may impair the quality of life of an individual. In some cases, in the absence of medical intervention, it could be life-threatening.^1^ Epilepsy occurs in all age groups, and it is considered to be one of the most debilitating neurological disease conditions globally.^2^ According to the World Health Organization’s (WHO) estimate, around 50 million people are affected by epilepsy globally, and nearly 80% (40 million) live in low- and middle-income countries.^2^ At present, the number of people living with epilepsy in the African continent is unknown, as a result of marked variations in prevalence data between and within countries, which is a consequence of poor record-keeping and the use of different diagnostic and recruitment protocols by epidemiology researchers in different countries.^3^ According to a 2004 WHO African region report, approximately 10 million people of all ages were affected by epilepsy in the African continent.^4^ The management of patients with epilepsy requires a long-term commitment from the general practitioner and/or specialist, carer and/or family and the patient.^1^ While in most cases, the use of antiepileptic drugs is considered to be the mainstay of treatment, using self-reporting diaries has been considered an important tool in the long-term management of epilepsy.^5,6^

Seizure diaries are a type of patient-reported outcome that is used to record seizure activity in the day-to-day life of the patient, to obtain insight into patients’ seizure triggers and events that may affect seizures, to monitor medication side effects and promote medication adherence and to communicate with the healthcare provider.^5,7,8^ The seizure diary requires the patients to self-report in the diary their seizure occurrence, duration of seizures, types of seizures and the day of occurrence of the seizures. Caregivers can also assist with diary entries.^5^ Seizure diaries are currently available in two major formats: paper and electronic. The paper-based diary is a basic calendar asking questions about patients’ seizure types and frequency, which is the industry standard.^5^ Patients can indicate the days of the month they had seizures by circling the dates in the calendar. Alternatively, the electronic diary (e-diary) comes in a digital format that can be uploaded online. It is a more detailed and accurate diary with time-stamped patient entries. Information on patients’ seizure types, frequency, duration, triggers, mood, medications and side effects can be logged with graphical reports and tabular summaries available to patients, caregivers and healthcare workers.^5^

Electronic-based seizure diaries are the most widely used and are reported to have several potential advantages over paper-based diaries.^5^ Despite their reported value in monitoring seizures and managing epilepsy, little information exists about the accuracy or validity of these tools or current norms and practices guiding the use of seizure diaries for managing epilepsy. In the present study, current international norms and practices were examined guiding the use of seizure diaries in the management of epilepsy and their implications for the management of epilepsy in Africa.

## Methods

A scoping review was conducted for the purpose of this study in order to explore the breadth of the literature and evidence available on the research topic, to summarise the evidence and to inform future research on the topic.^9^ In order to take a structured approach to investigating the current norms and international practices that guide the use of seizure diaries in the management of epilepsy, this review used the five-point framework for data interrogation and analysis proposed by Arksey and O’Malley, namely (1) identify research questions, (2) identify relevant sources or studies, (3) select relevant literature, (4) chart data and (5) collate and analyse the literature.^10^ We found no previously published scoping review protocol for this review or the topic of interest. Hence, a protocol was developed for the study.

### Identification of research questions

This review aimed to identify a wide range of literature relating to the use of seizure diaries for the management of epilepsy. Based on the initial literature search results, a research question was formulated. The main research question that guided this study is as follows: ‘what are the current norms and international practices guiding the use of seizure diaries in the management of epilepsy?’

### Identification of relevant sources or studies

An initial search was conducted using Medical Literature Analysis and Retrieval System Online (MEDLINE) and Cumulative Index to Nursing and Allied Health Literature (CINAHL), with the aim of identifying common keywords or phrases in the titles and abstracts of retrieved and relevant articles. Identified keywords or phrases were then used to develop full-search strategies adapted for each information source. Reference lists of sources included were reviewed to determine if potential sources had been overlooked by the search strategy. A literature search was conducted on the following databases: EBSCOhost, Scopus, Cochrane, MEDLINE, CINAHL, Web of Science, Academic Search Ultimate, Africa-Wide Information, American Psychological Association (APA) PsycArticles, APA PsycInfo, Centre for Agriculture and Bioscience (CAB) Abstracts, Communication & Mass Media Complete, Educational Research Information Center (ERIC), Health Source – Consumer Edition, Health Source: Nursing/Academic Edition, Humanities Source Ultimate and Sociology Source Ultimate. Keyword entries used were ‘epilepsy diary’, ‘seizure diary’, ‘fit charts’, ‘seizure records’, ‘paper diaries’, ‘seizure tracker’ and ‘international updates on epilepsy’. Keywords were used singly or in combination. Grey literature was examined using Google and Google Scholar search engines in an attempt to identify any unpublished studies relevant to the research question. Identified citations were imported into EndNote X9 (Clarivate Analytics, Chandler, Arizona, United States).^11^ Articles were initially screened for inclusion after a quick review of the title and abstracts against the inclusion criteria. If doubt existed regarding its suitability, the article’s full text was retrieved and reviewed for possible inclusion. If doubt still existed and reviewers could not reach an agreement, an independent colleague was consulted for deliberation. The first search was conducted on 22 February 2021; this was followed by a serial search every four months for any recent publication on the topic of interest. The last search was conducted on 31 December 2021.

### Selection of relevant literature

This scoping review included articles, randomised clinical trials, reviews and conference proceedings published in English since 1981, from any geographical location, as long as it related to the use of seizure diaries for clinical management of epilepsy and seizure monitoring (Table 1). The concepts of interest were standards for an ideal seizure diary, models or types of seizure diaries currently used in clinical practice, how diaries are being used to manage epilepsy and the efficacy of diaries for patient management. Sources of evidence were quantitative and qualitative research that reported on using diaries or seizure monitoring charts for the management of epilepsy and published in peer-reviewed academic journals and verifiable sites or web pages. A search was made on electronic databases, such as EBSCOhost, Scopus, Cochrane and Web of Science, for published articles and relevant reviews that addressed this study’s research question. The inclusion and exclusion criteria are presented in Table 1.

**TABLE 1:** Inclusion and exclusion criteria.

Inclusion criteria	Rationale
Study type	Human studies
	Qualitative studies
	Quantitative studies
	Randomised clinical trials
	Experimental studies
Year	Studies published from 1981 to 2021
Language	English
Location	Any geographical location
Publication type	Research articles
	Reviews
	Randomised clinical trials
	Conference proceedings
	Published in peer-reviewed journals and verified online reports or web pages
	Unpublished studies relevant to the topic
Context	Literature discussing the use of diaries or seizure records for managing epilepsy or seizures
**Exclusion criteria**	
Study type	Nonhuman studies
Language	Reports not published in English
Publication type	Non-peer-reviewed studies
	Social media reports
	Unverified reports
Context	Studies not discussing the use of diaries to manage patients with epilepsy.

**FIGURE 1:**
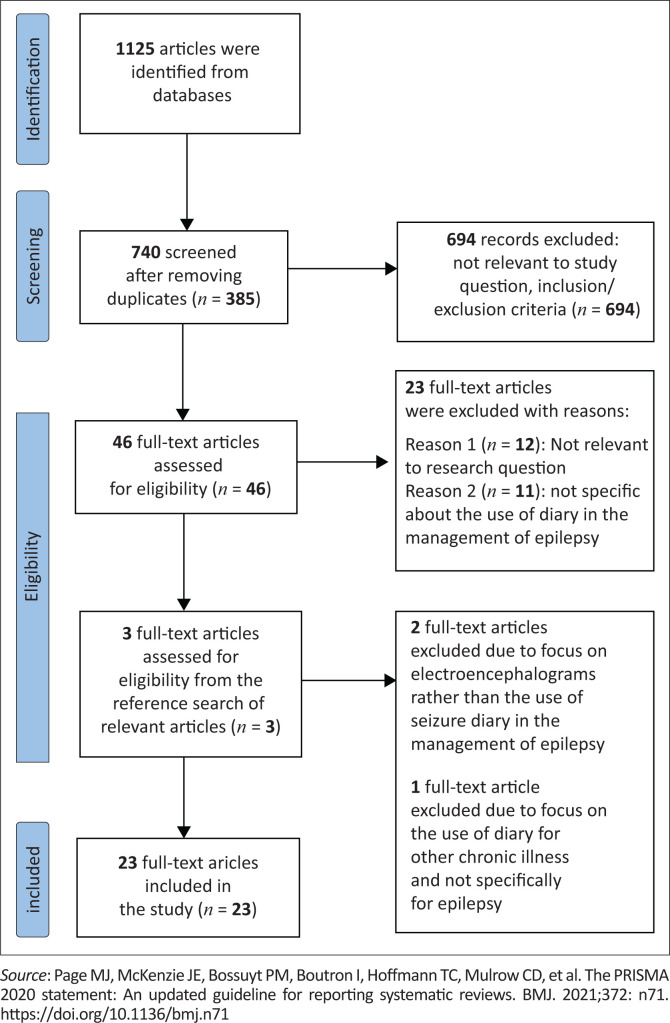
Flow chart of article selection.

The selection process provided the basis for identifying 1125 potentially relevant sources. After removing duplicate articles (*n* = 385), the lead author screened the titles and abstracts for eligibility. A total of 694 articles were rejected because of a lack of relevance to the subject. All authors reviewed the remaining 46 articles, and 23 articles were agreed on for inclusion. The selection process is presented in Figure 1.

### Charting of data

Each article included in the search was summarised and is presented in Table 2 under the following headings: authors, title of article, journal, author location, research design, sample size and comments relevant to the use of seizure diary.

**TABLE 2:** Overview of articles used in this review.

Authors	Title of articles	Journal	Author location	Research designs	Sample size (*n*)	Authors’ comments about the diaries
Blachut et al.^13^	Counting seizures: The primary outcome measure in epileptology from the patients’ perspective	*Seizure*	Germany	Retrospective survey	170	Almost two-thirds of patients in this study reported keeping seizure diaries. Patients appeared to know that they underreported seizures, which reduced the validity of patient seizure counts.
Blachut et al.^14^	Subjective seizure counts by epilepsy clinical drug trial participants are not reliable	*Epilepsy and Behavior*	Germany	Prospective (noninterventional) study	100	Epilepsy patients who participate in clinical trials underreport seizures, as do patients in general.
Corey et al.^15^	The accuracy of the self-reported history of seizures in Danish, Norwegian and US twins	*Epilepsy Research*	Denmark, Norway, US	Retrospective survey	47 626	The accuracy of self-reported epilepsy and febrile seizures was high across all populations in this study.
Detyniecki et al.^16^	Prevalence and predictors of seizure clusters: A prospective observational study of adult patients with epilepsy	*Epilepsy and Behavior*	US	Prospective (observational) study	247	Patients may have been more or less likely to accurately document information in seizure diaries, depending on seizure burden.
Fisher et al.^5^	Seizure diaries for clinical research and practice: Limitations and future prospects	*Epilepsy and Behavior*	US	Narrative review	0	Diary-based observational studies have the advantage of low cost, allowing locus of control by the patient, and testing in a real-world environment. It is useful as a descriptive snapshot of a population.
Ernst et al.^7^	Medication adherence in women with epilepsy who are planning pregnancy	*Epilepsia*	US	Prospective (multicentre observational) study	86	Diary-compliant patients reported a high rate of anti-epilepsy medication adherence.
Ferastraoaru et al.^17^	Characteristics of large patient-reported outcomes: Where can one million seizures get us?	*Epilepsia Open*	US	Longitudinal (observational) study	10 186	Using electronic patient-reported seizure diary databases presents challenges and limitations, of which the most important is the reliability of data. Reported seizures may be nonepileptic, overreported or underreported.
Fisher et al.^18^	Use of an online epilepsy diary to characterise repetitive seizures	*Epilepsy and Behavior*	US	Descriptive (observational) survey	5098	A limitation of the study was the observational and uncontrolled nature of the data and subjective reporting of events.
Fisher et al.^19^	Tracking epilepsy with an online seizure diary	*Acta Paediatrica*	US	Narrative review	0	After thousands of users have entered data longitudinally into the diary, it will become possible to make observations on patterns.
Glueckauf et al.^20^	Consistency of seizure frequency estimates across time, methods, and observers	*Health Psychology*	US	Mixed methods (retrospective and prospective *study*)	32	The reliability of self-reporting seizures using the recall or prospective diary methods is high.
Goldenholz et al.^21^	Is seizure frequency variance a predictable quantity?	*Annals of Clinical and Trans-lational Neurology*	US	Longitudinal (predictive *survey*)	3124	By using data from three independently collected patient diary databases, variance in seizure frequency was predictable, based on knowledge of the mean seizure frequency.
Hall et al.^22^	Early follow-up data from seizure diaries can be used to predict subsequent seizures in same cohort by borrowing strength across participants	*Epilepsy and Behavior*	US	Prospective study	71	Three models were developed using 30 days of nightly seizure diary data in 71 patients to predict subsequent seizures in the same patients over 30 days. The use of paper-based diaries was a limitation because of the absence of time stamping.
Haut et al.^23^	Seizure occurrence: Precipitants and prediction	*Neurology*	US	Prospective study	71	In a paper-based diary study, seizure prediction based on precipitants, premonitory features and self-prediction may provide a foundation for pre-emptive treatment.
Haut et al.^24^	Modeling seizure self-prediction: An e-diary study	*Epilepsia*	US	Prospective study	19	Seizure self-prediction is possible for a subgroup of patients with epilepsy in an e-diary study.
Haut et al.^25^	Clinical features of the pre-ictal state: Mood changes and premonitory symptoms	*Epilepsy and Behavior*	US	Prospective study	19	Diary studies rely entirely on self-reports. Lack of patient accuracy in self-reporting may limit reliability.
Haut et al.^26^	Predicting seizures: A behavioral approach	*Neurologic Clinics*	US	Predictive study	N/A	Analysis of data from paper and electronic diaries suggests that patient seizure prediction is feasible.
Hoppe et al.^27^	Epilepsy: Accuracy of patient seizure counts	*Archives of Neurology*	Germany	Randomised controlled trial	91	Patient seizure counts are not valid information.
Illingworth et al.^28^	A method for identifying associations between seizures and possible trigger events in adults with intellectual disability	*Epilepsia*	United Kingdom	Prospective study	5	The study’s limitation was the unreliability of data collected using a pen- and paper-based diary.
Karoly et al.^29^	Are the days of counting seizures numbered?	*Current Opinion in Neurology*	Australia	Narrative review	0	Diaries are highly unreliable; nevertheless, manual diaries are exclusively relied on for clinical trials.
Milton et al.^30^	Timing of seizure recurrence in adult epileptic patients: A statistical analysis	*Epilepsia*	Canada	Prospective study	24	Seizures reoccur in patients randomly and may involve factors such as stress, having missed nights of sleep, skipping medications or consuming alcohol.
Le et al.^31^	An online diary for tracking epilepsy	*Epilepsy and Behavior*	US	Descriptive study	1944	Anonymous data from diaries provided a snapshot of the characteristics of a segment of the epilepsy community.
Neugebauer^32^	Reliability of seizure diaries in adult epileptic patients	*Neuroepi-demiology*	US	Prospective study	54	The daily diary is a reliable method for securing data on seizure counts.
Poochikian-Sarkissian et al.^33^	Patient awareness of seizures as documented in the epilepsy monitoring unit	*Canadian Journal of Neuro-science Nursing*	Canada	Prospective study	138	Incomplete data in seizure diaries is probably a widespread problem and may have an important impact on treatment, safety and quality of life.

US, United States; N/A, not applicable.

### Ethical considerations

Ethical clearance to conduct the study was obtained from the Health Sciences Research Ethics Committee of the Faculty of Health Sciences at the University of the Free State (ref. no. UFS-HSD2020/1385/2411).

## Results

As reported in Table 2, of the 23 articles, almost half (48%; *n* = 11) were prospective studies (Table 2). The majority (65.2%; *n* = 15) were studies conducted in the United States of America, while 21.7% (*n* = 5) of the studies were conducted in Europe (Denmark 1, Germany 3 and the United Kingdom 1). No article of relevance was found for a study conducted in Africa, suggesting that very limited research has been conducted on the use of seizure diaries as a tool for managing epilepsy in Africa. An analysis of the publication years of studies revealed that the majority (87%; *n* = 20) had been published between 2006 and 2018, while three (13%) had been published between 1987 and 1990.

### Themes identified

Identified themes, categories and subcategories are presented in Table 3.

**TABLE 3:** Identified themes, categories and subcategories.

Themes	Categories	Subcategories	References
Types of seizure diaries used in clinical practice	Paper-based diary	Advantages of a paper-based diary	5, 34
		Disadvantages of a paper-based diary	5, 19, 28, 31
	Electronic diary	Advantages of an electronic diary	5, 18, 19, 25, 31
		Disadvantages of an electronic diary	5
Contents and structure of a standardised seizure diary	Seizure types	–	5, 18, 31, 35, 36
	Seizure frequency	–	5, 13, 14, 16, 17, 18, 20, 21, 27, 29, 31, 33
	Days of seizure occurrence	–	5, 18, 19, 30, 31
	Others	Seizure duration	5, 17, 18, 31
		Time of seizure	13, 14, 18, 19, 27, 31
		Seizure triggers	5, 18, 31
		Seizure clusters	16, 18, 37, 38
		Medication history	5, 18
The use and efficacy of seizure diaries in medicine	Use of seizure diary in clinical practice	–	5,13,14,19,36,39
	Use of seizure diary for clinical research	–	7, 16, 17, 21, 22, 24, 25, 26, 27, 30, 31
Challenges facing the use of seizure diaries for patient management	Patients’ compliance	–	7, 16, 20, 24, 27
	Reliability of patient-reported seizures	–	13, 14, 16, 18, 28
	Validity of patient-reported seizures	–	7, 14, 17, 22, 23

## Discussion

### Theme 1: Types of seizure diaries used in clinical practice

The use of patient diaries to assist with clinical management and patient care is a standard practice that has been incorporated into medical practice over the years.^40,41,42^ Diaries have been used to document symptoms and factors that may have precipitated the symptoms, patient responses to symptoms, efficacy of treatment response and medication adherence.^40,41,42^ Diaries are regarded as the backbone of clinical epilepsy management and therapeutic trials.^5^ The findings of the review by Fisher et al.^5^ revealed that seizure diaries are available in two formats, namely paper-based diaries and e-diaries.

#### Category 1.1: Paper-based diary

A paper-based diary is a hard copy document that is used to record information such as patients’ seizure types and frequency.^5^ This type of seizure diary requires the patient or caregiver to manually write in the required information. A paper-based diary is the preferred choice in a resource-limited setting where patients have limited access to the Internet or electronic devices.^5,34^

**Subcategory 1.1.1: Advantages of a paper-based diary:** Documented advantages of the paper-based diary are that they are cheaper to create, as they do not require Internet access or expensive devices; it is easier for patients to learn how to use; it is easier to use because it does not require great intellectual capabilities or specialised skills (i.e. computer skills) to complete.^5^ In addition, a paper-based diary can be successfully used by a predominantly illiterate community and can easily be made available to patients.^34^

**Subcategory 1.1.2: Disadvantages of a paper-based diary:** The disadvantages of a paper-based diary include that it can easily be lost, misplaced or not brought along by patients to clinic visits^5,31^ and that patient data cannot be backed up or reconstructed if the diary is lost.^5^ Paper-based diaries can be completed retrospectively, and it is prone to recall bias, which calls into question the validity of patient-reported data.^28^ Written data are difficult to transform into electronic data, making it difficult to visualise trends and relationships between listed factors.^19^

#### Category 1.2: Electronic diary

In contrast to paper-based diaries, an e-diary can be accessed via different online browsers on devices such as computers, laptops and smartphones.^5,41^ An e-diary can record more detailed data than a paper-based diary. Among its various features are that programming can improve data validity, real-time transmission of patient information is possible and reminders can be sent to subjects.^5,43^

**Subcategory 1.2.1: Advantages of an electronic diary:** The literature reports several advantages of using an e-diary. An online database serves as a safe storage site for storing patients’ information anonymously.^18^ An e-diary is not prone to being easily lost or misplaced,^19,31^ and data can be made available in graphical or tabular format for easy visualisation of trends.^31^ Electronic diaries are easily accessible via handheld devices like smartphones and computers.^5^ The e-diary allows for precise time recording; an entry is time-stamped and it is difficult to backfill or complete retrospectively.^25^ The e-diary can be adapted to prevent information entered into the diary from being edited.^25^ An e-diary can be programmed to provide reminders to improve patient compliance.^19^ Large online databases to which patients contribute information can serve as a rich data pool for authorised researchers.^5^

**Subcategory 1.2.2: Disadvantages of an electronic diary:** Reported disadvantages of electronic diaries are that an e-diary requires the user to be technologically proficient and to have access to electronic devices – which patients cannot always afford.^5^ It may be difficult to use an e-diary for patients who are children, as it will require a caregiver’s input, which may be problematic because of the presence of multiple caregivers.^5^ If multiple caregivers have access to the diary, it may compromise patients’ privacy.^5^

### Theme 2: Contents and structure of an ideal seizure diary

While there is little empirical evidence or a theoretical foundation to inform the content of patient diaries, the design and contents of most patient diaries are tailored to achieve the goal of clinical management.^41^ In the design of diaries for clinical drug trials, one type of diary will not be appropriate for all clinical studies because of the inconsistency of the questions posed. Trying to predict all possible data will lead to requesting too much information, which may make the diary impractical to use. The best diaries ask only what is needed with an efficient and user-friendly design.^5^ The findings of the present study revealed that a standardised seizure diary must contain three vital, basic pieces of information, namely seizure type, seizure frequency and days of seizure occurrence.^5^

#### Category 2.1: Seizure type

The management of epilepsy recognises various seizure types, which may affect different age groups. Children, more than adults, have been reported to present with multiple seizure types.^5^ Treatment is often tailored to consider seizure types; hence, accurate description and categorisation of seizure types are important for proper management. To this end, some researchers^5,18,31^ consider information on seizure type to be an essential component of a seizure diary. The International League Against Epilepsy (ILAE) provides a detailed operational classification of seizures.^35^ Fisher et al.^5^ suggested that seizure types may be recorded using codes such as A, B and C to represent different types of seizures known to the diary developers and the patients.^5^ Other researchers suggest writing out the common types of seizures in full if the space is available in the diary.^36^

#### Category 2.2: Seizure frequency

Seizure frequency is another important piece of information that must be captured in a seizure diary, as reported by most of the literature reviewed.^5,13,14,16,17,18,20,21,27,31,33^ Patients document their seizure occurrence, which can be used in estimating the seizure frequency. The estimation of seizure frequency is a cornerstone of clinical epilepsy management and evaluation of new therapies.^29^ Self-reported seizure counts of patients with epilepsy guide treatment decisions and are often the primary outcome measure of clinical trials in epilepsy.^14,27^ Patients’ medication dosages and clinic visit frequency are adjusted according to the trends of patient-reported seizure frequencies.^33^ Some scholars^16,18^ reported that information on seizure frequency could be used to identify seizure clusters.^37^ Karoly et al.^29^ reported that seizure counts remain the primary way of quantifying patients’ epilepsy. Seizure counting does not require specialised equipment or tests and is entrusted to patients or caregivers, commonly using a seizure diary.^29^

#### Category 2.3: Days of seizure occurrence

Days of seizure occurrence is one of the three most important pieces of information that must be captured in a standardised seizure diary.^5,18,19,30,31^ Researchers suggest that a seizure diary must contain a suitable calendar for charting days with seizures in order to eliminate patient recall bias.^5,18,19,30,31^ Calendars are available as hard copy (paper-based diary) or electronic calendars (e-diary). Several events can be recorded on a calendar diary, such as seizures, mood, menstruation, medications and side effects, by day or time of day.^18,19^

#### Category 2.4: Other information

Additional information that can be recorded in the seizure diary includes seizure duration, time of seizure, seizure triggers, seizure clustering, medication regimens, missed medications, medication side effects and patient mood. A seizure diary may include the duration of seizures as additional information provided by the user of the diary and caregiver. Authors of the reviewed literature^5,17,18,31^ described seizure duration as additional information that could be provided by users as part of patient history. Prolonged seizure duration increases the risk of progression to status epilepticus, which has been reported to account for increased morbidity and mortality of patients with epilepsy.^44^ Time of the seizure can be recorded in a seizure diary.^13,14,18,19,27,31^ Seizures can occur in the daytime or at night. Night-time seizures are more likely to be undocumented by patients because of unawareness.^13,27^ Seizure triggers are additional information that can be recorded in the seizure diary.^5,18,31^ Some patients are familiar with events that may trigger their seizures, but it is common for a patient not to know what triggered a seizure. Successfully identifying triggers can help decrease seizure frequency in patients. Some authors of the reviewed literature reported using a seizure diary to monitor common seizure triggers.^16,17,23,24,25,26^ Stressful life events, mood changes, missed or changed medications, altered sleep, alcohol consumption, menstruation, anxiety, bright or flashing lights and constipation are some of the seizure precipitants commonly reported in the literature.^17,18,22,25,28,30,31^ Seizure clusters are patterns of seizures that occur multiple times a day,^18^ although there is no consensus on the definition of seizure clusters.^18,37^ Detyniecki et al.^16^ defined seizure clusters as two or more seizures in 6 h, while Haut et al.^38^ used an alternative definition, defining seizure clusters as three or more seizures in any given 24-h period. Fisher and colleagues reported that seizure clusters are additional information that can be ascertained from recorded data in a seizure diary.^5,19^ Diary data that include seizure time from online electronic seizure diaries, such as Nile (Nile AI, Inc., Los Angeles, California, United States) and Seizure Tracker (Seizure Tracker LLC, Springfield, Virginia, United States), are used to identify repetitive seizure patterns that signify clusters in patients with epilepsy.^17,18^ People with high daily seizure counts are likely to experience clusters every day.^18^ Medication regimens can be recorded as additional details in seizure diaries.^5,18^ The ‘best dose’ of medication is the one that controls seizures and is associated with the least side effects.^43^ Information on other chronic medications used for medical conditions apart from epilepsy may be vital in order to avoid medication errors because of drug–drug interactions.^45^ Information on medication adherence is important for seizure control, and daily diary inputs may act as reminders to promote medication adherence.^23^ Ernst et al.^7^ found seizure diaries to be helpful in tracking patients’ medication adherence in a subset of women with epilepsy who were planning pregnancy. Some electronic diaries allow users to document their medication names and doses; users can also indicate if they missed medications or took extra doses.^18,19^

### Theme 3: The use and efficacy of seizure diaries in medicine

Information synthesised from the reviewed literature reveals that seizure diaries are used in two major forms: clinical practice and research.

#### Category 3.1: Using seizure diaries in clinical practice

A seizure diary is a self-management tool for patients living with epilepsy.^8,39^ Some researchers^13,14^ reported on using paper-based diaries for the clinical management of patients with epilepsy. Although prone to recall and response bias, a basic paper-based diary can provide a history of a patient’s seizure types, seizure frequency and the calendar dates of seizure occurrence.^5,19^ A paper-based diary can be used as a source of patient information that is useful when patients migrate to other locations or when a lack of continuity regarding healthcare providers causes patients to see a different doctor every time they visit a clinic. Electronic diaries are online applications for patients living with epilepsy.^39^ Examples of popular web-based diaries include Nile (formerly My Seizure Diary, available at https://www.epilepsy.com/)^39^ and Seizure Tracker (available at https://seizuretracker.com/).^17,36^ Both diaries allow patients and caregivers to log seizures of different types, medication dosages, events by time, seizure duration, missed medication, additional medication and medication side effects, and they provide graphical and tabular summaries of information that can be assessed by a medical team via the physician portal or e-mail, if permitted.^5^

The use of seizure diaries in predicting seizures was reported by six researchers.^21,22,23,24,25,26^ Haut et al.,^25^ in a study using the e-diary, reported that changes in mood such as happiness, sadness and nervousness and premonitory features such as blurred vision, dizziness and light sensitivity contribute to the prediction of seizures over 12 h. In another study using an electronic diary, Haut et al.^24^ reported that 9 of 19 (43%) participants with epilepsy were able to accurately predict their seizures, drawing on awareness of mood and premonitory symptoms.

#### Category 3.2: Using seizure diaries for clinical research

Diaries can be used for clinical research; almost all the studies reviewed commented on the use of seizure diaries for clinical research.^7,16,17,21,22,24,25,26,27,30,31^ Seizure diaries could be used in investigator-supervised research, such as randomised controlled trials of novel epilepsy therapies, prospective observational studies, or unsupervised studies, such as *ad hoc* analysis of self-reported anonymous diaries.^31^ Longitudinal data entered into seizure diaries can be used to make observations, and observational data can be used to generate hypotheses to be tested in clinical trials.^19^ In addition, information such as demographic data, seizure types, seizure precipitants and medication usage that is captured in diary-based studies can be used to develop descriptive snapshots of the population.^31^

### Theme 4: Challenges related to using seizure diaries for patient management

#### Category 4.1: Patients’ compliance

Compliance means to adhere to something; clinicians expect patients to comply regarding diary use. In this scoping review, we found that compliance was often discussed as one of the major challenges relating to the use of seizure diaries for patient management.^7,13,14,16,19,20,22,23,24,26,27^ Nevertheless, patient-reported compliance was high in most of the literature reviewed. Blachut et al.^13^ stated that the majority of patients in their study reported keeping a seizure diary, most keeping the diary themselves, and in some cases, patients were assisted. The high level of compliance documented by these studies may be the result of various strategies used by the researchers to enhance compliance. Documented strategies to enhance patient compliance include sending electronic reminders^7,16,20,24,27^ and training patients on how to use the diary.^23,25,28^ Despite these strategies, Hoppe et al.^27^ reported that in their study, reminding participants did not enhance seizure documentation.^27^ It is also reported that severe epilepsy, such as drug-resistant seizures and increased seizure frequency, could enhance compliance with diary use as opposed to patients with well-controlled epilepsy who may have little motivation to utilise the diary.^17,31^

Keeping a seizure diary empowers the patient to be actively involved in controlling their health. Maintaining a diary may help improve compliance with treatment.^7^ Blachut et al.^14^ stated that patients are compliant with documentation if they feel it is important for monitoring their disease and may affect their treatment.

Furthermore, healthcare practitioners’ interest in the patients’ seizure diary has been suggested to improve patient compliance with diary use. Blachut et al.^13^ reported a strong positive correlation between patients’ commitment to seizure documentation and doctors’ behaviour. Doctors taking time to review the diary reinforced positive diary behaviour by patients,^13^ which should be encouraged to improve patient compliance.

#### Category 4.2: Reliability of data on patient-reported seizures

Data on seizures, self-reported using either paper-based or electronic diaries, have long been relied upon in clinical drug trials and clinical management of patients with epilepsy. The accuracy of subjective self-reporting continues to be debated.^23^ Thirteen of the studies reviewed described the accuracy of patient-reported seizures as being a limitation in their studies.^13,14,16,18,28^ Some researchers suggested that inaccuracies in seizure reporting were because of patients’ inability to recognise their seizures (seizure unawareness), which leads to underreporting.^13,14,27^ Poochikian-Sarkissian et al.^33^ reported that patients in their study recognised only 44.5% of complex partial seizures and generalised tonic-clone seizures, thus leading to questions about the reliability of patients’ diary-reported seizures. These findings are supported by other researchers.^46,47^ Patients also complete their diaries retrospectively, thus affecting the reliability of the data.^7,23^

In contrast, three of the studies reviewed found patients’ self-reported seizure estimates to be reliable and relevant.^15,20,32^ Glueckauf et al.^20^ evaluated the consistency of seizure frequency estimates among patients and caregivers. The study reported that patients provide more reliable seizure frequency estimates than caregivers. Neugebauer^32^ reported that using the diary in clinical settings still enjoys high reliability in dedicated patients. Corey et al.^15^ reported that the accuracy of self-reporting of epilepsy and febrile seizures was high among patients providing health histories in all population groups studied. Detynieki et al.^16^ reported that following up paper-based diary patients, collecting and reviewing the diary at regular intervals (electronic diaries being reviewed on a monthly basis) and comparing the provided information with diary entries help ensure the accuracy of data. However, most researchers described self-reported diaries as being inaccurate and unreliable. Karoly et al.^29^ stated that using video electroencephalogram (EEG) is a more accurate and modern technique of monitoring and reporting seizures. However, this technique may not be suitable for all patients and clinics because of affordability. Patients are less likely to accept a more invasive monitoring technique in exchange for more accurate information.

#### Category 4.3: Validity of data on patient-reported seizures

Subjective data reflect patients’ reported perspectives via their seizure diaries, while objective data reflect verifiable facts using clinical events correlated to electrographic seizures.^29^ Seizures can be verified using video EEG, ambulatory EEG, implantable recording devices, smartwatches and other modern devices.^5,36,48,49^ Researchers reported that patient-reported seizure diary data are subjective and not verifiable.^7,14,17,22,23^ Patient-reported, subjective data are prone to inaccuracies and being unreliable. Some of the difficulties experienced in using objective methods in validating seizure diary data, as reported by some researchers, include problems with electrode placement, the high cost of EEG equipment, safety concerns and practical difficulties.^20,28^ Despite these difficulties, some studies reviewed validated diary data.^15,21,27,33^ These authors reported that combining diary data with EEG readings can help validate diary data.

## Limitations

A limitation of this study is the paucity of literature on the use of the seizure diary in epilepsy management in low-income countries of the world in general and the African continent in particular. More information would have enabled the documentation of trends in the use of seizure diaries in managing epilepsy in a resource-poor setting.

## Conclusion

This scoping review showed that seizure diaries remain relevant for epilepsy management in a resource-poor setting where modern devices for objective detection and recording of seizures are unaffordable. Without feasible alternatives, affordable seizure diaries are essential for monitoring epilepsy in a resource-poor setting, despite its challenges. Electronic- and paper-based diaries are the two forms of diaries in use. The high cost of data and the expense of devices required to access an e-diary make this form unsuitable for resource-limited settings.^48,49,50^ Patient compliance and the reliability and validity of patient-reported data are some of the challenges associated with the use of seizure diaries in clinical practice. Training patients to use the seizure diary, encouraging them to complete the diary as soon as possible after a seizure and motivating them to use the diary to keep track of seizures, which influences their treatment, may help improve the use of the seizure diary.

## References

[1] Smith D, Chadwick D. The management of epilepsy. J Neurol Neurosurg Psychiatry. 2001;70(suppl. 2):ii15–ii21. 11385045 10.1136/jnnp.70.suppl_2.ii15 PMC1765559

[2] World Health Organization. Epilepsy [homepage on the Internet]. 2019 [cited 2021 Feb 17]. Available from: https://www.who.int/news-room/fact-sheets/detail/epilepsy

[3] Owolabi LF, Adamu B, Jibo AM, et al. Prevalence of active epilepsy, lifetime epilepsy prevalence, and burden of epilepsy in sub-Saharan Africa from meta-analysis of door-to-door population-based surveys. Epilepsy Behav. 2020;103:106846. 10.1016/j.yebeh.2019.106846 31941583

[4] World Health Organization. Epilepsy in the WHO African region: Bridging the gap [homepage on the Internet]. 2004 [cited 2022 Jan 10]. Available from: https://www.ecoi.net/en/file/local/1320358/432_1198069054_epilepsy-in-african-region.pdf

[5] Fisher RS, Blum DE, DiVentura B, et al. Seizure diaries for clinical research and practice: Limitations and future prospects. Epilepsy Behav. 2012;24(3):304–310. 10.1016/j.yebeh.2012.04.128 22652423

[6] Egerod I, Schwartz-Nielsen KH, Hansen GM, Lærkner E. The extent and application of patient diaries in Danish ICUs in 2006. Nurs Crit Care. 2007;12(3):159–167. 10.1111/j.1478-5153.2007.00219.x 17883648

[7] Ernst LDL, Harden CL, Pennell PB, et al. Medication adherence in women with epilepsy who are planning pregnancy. Epilepsia. 2016;57(12):2039–2044. 10.1111/epi.13586 27778312 PMC6374285

[8] Epilepsy Foundation. My seizure diary [homepage on the Internet]. 2019 [cited 2022 Aug 1] Available from: https://www.epilepsy.com/living-epilepsy/epilepsy-foundation-my-seizure-diary

[9] Tricco AC, Lillie E, Zarin W, et al. A scoping review on the conduct and reporting of scoping reviews. BMC Med Res Methodol. 2016;16(1):1–10. 10.1186/s12874-016-0116-4 26857112 PMC4746911

[10] Arksey H, O’Malley L. Scoping studies: Towards a methodological framework. Int J Soc Res Methodol. 2005;8(1):19–32. 10.1080/1364557032000119616

[11] Windows. Analytics C. The EndNote guided tour: Windows [homepage on the Internet]. 2018 [cited 2022 Feb 22]. Available from: http://download.endnote.com/training/Little%20Book/EndNote_X9_Guided_Tour-Windows.pdf

[12] Page MJ, McKenzie JE, Bossuyt PM, Boutron I, Hoffmann TC, Mulrow CD, et al. The PRISMA 2020 statement: An updated guideline for reporting systematic reviews. BMJ. 2021;372: n71. 10.1136/bmj.n71 33782057 PMC8005924

[13] Blachut B, Hoppe C, Surges R, Stahl J, Elger CE, Helmstaedter C. Counting seizures: The primary outcome measure in epileptology from the patients’ perspective. Seizure. 2015;29:97–103. 10.1016/j.seizure.2015.03.004 26076850

[14] Blachut B, Hoppe C, Surges R, Elger C, Helmstaedter C. Subjective seizure counts by epilepsy clinical drug trial participants are not reliable. Epilepsy Behav. 2017;67:122–127. 10.1016/j.yebeh.2016.10.036 28139449

[15] Corey LA, Kjeldsen MJ, Solaas MH, Nakken KO, Friis ML, Pellock JM. The accuracy of self-reported history of seizures in Danish, Norwegian and US twins. Epilepsy Res. 2009;84(1):1–5. 10.1016/j.eplepsyres.2008.11.014 19128944 PMC2674277

[16] Detyniecki K, O’Bryan J, Choezom T, et al. Prevalence and predictors of seizure clusters: A prospective observational study of adult patients with epilepsy. Epilepsy Behav. 2018;88:349–356. 10.1016/j.yebeh.2018.09.035 30344026

[17] Ferastraoaru V, Goldenholz DM, Chiang S, Moss R, Theodore WH, Haut SR. Characteristics of large patient-reported outcomes: Where can one million seizures get us? Epilepsia Open. 2018;3(3):364–373. 10.1002/epi4.12237 30187007 PMC6119749

[18] Fisher RS, Bartfeld E, Cramer JA. Use of an online epilepsy diary to characterize repetitive seizures. Epilepsy Behav. 2015;47:66–71. 10.1016/j.yebeh.2015.04.022 26046724

[19] Fisher RS. Tracking epilepsy with an electronic diary. Acta Paediatr. 2010;99(4):516–518. 10.1111/j.1651-2227.2010.01694.x 20105139

[20] Glueckauf RL, Girvin JP, Braun JR, Bochen JL. Consistency of seizure frequency estimates across time, methods, and observers. Health Psychol. 1990;9(4):427. 10.1037/0278-6133.9.4.427 2115437

[21] Goldenholz DM, Goldenholz SR, Moss R, et al. Is seizure frequency variance a predictable quantity? Ann Clin Transl Neur. 2018;5(2):201–207. 10.1002/acn3.519 PMC5817844 29468180

[22] Hall CB, Lipton RB, Tennen H, Haut SR. Early follow-up data from seizure diaries can be used to predict subsequent seizures in same cohort by borrowing strength across participants. Epilepsy Behav. 2009;14(3):472–475. 10.1016/j.yebeh.2008.12.011 19138755 PMC4283490

[23] Haut SR, Hall CB, Masur J, Lipton RB. Seizure occurrence: Precipitants and prediction. Neurology. 2007;69(20):1905–1910. 10.1212/01.wnl.0000278112.48285.84 17998482

[24] Haut SR, Hall CB, Borkowski T, Tennen H, Lipton RB. Modeling seizure self-prediction: An e-diary study. Epilepsia. 2013;54(11):1960–1967. 10.1111/epi.12355 24111898 PMC3833277

[25] Haut SR, Hall CB, Borkowski T, Tennen H, Lipton RB. Clinical features of the pre-ictal state: Mood changes and premonitory symptoms. Epilepsy Behav. 2012;23(4):415–421. 10.1016/j.yebeh.2012.02.007 22424857

[26] Haut SR, Lipton RB. Predicting seizures: A behavioral approach. Neurol Clin. 2009;27(4):925–940. 10.1016/j.ncl.2009.06.002 19853216

[27] Hoppe C, Poepel A, Elger CE. Epilepsy: Accuracy of patient seizure counts. Arch Neurol. 2007;64(11):1595–1599. 10.1001/archneur.64.11.1595 17998441

[28] Illingworth JL, Watson P, Xu S, Manford M, Ring H. A method for identifying associations between seizures and possible trigger events in adults with intellectual disability. Epilepsia. 2015;56(11):1812–1818. 10.1111/epi.13137 26385590

[29] Karoly P, Goldenholz DM, Cook M. Are the days of counting seizures numbered? Curr Opin Neurol. 2018;31(2):162–168. 10.1097/WCO.0000000000000533 29369115

[30] Milton JG, Gotman J, Remillard GM, Andermann F. Timing of seizure recurrence in adult epileptic patients: A statistical analysis. Epilepsia. 1987;28(5):471–478. 10.1111/j.1528-1157.1987.tb03675.x 3653049

[31] Le S, Shafer PO, Bartfeld E, Fisher RS. An online diary for tracking epilepsy. Epilepsy Behav. 2011;22(4):705–709. 10.1016/j.yebeh.2011.08.035 21975298

[32] Neugebauer R. Reliability of seizure diaries in adult epileptic patients. Neuroepidemiology. 1989;8(5):228–233. 10.1159/000110187 2812181

[33] Poochikian-Sarkissian S, Tai P, Del Campo M, et al. Patient awareness of seizures as documented in the epilepsy monitoring unit. Can J Neurosci Nurs. 2009;31(4):22–23. 20085117

[34] Wiseman V, Conteh L, Matovu F. Using diaries to collect data in resource-poor settings: Questions on design and implementation. Health Policy Plan. 2005;20(6):394–404. 10.1093/heapol/czi042 16183737

[35] Fisher RS, Cross JH, D’Souza C, et al. Instruction manual for the ILAE 2017 operational classification of seizure types. Epilepsia. 2017;58(4):531–542. 10.1111/epi.13671 28276064

[36] Seizure Tracker. Printable Seizure Log [homepage on the Internet]. [cited 2022 Mar 6]. Available from: https://seizuretracker.com/Seizure_Log_Printable.php

[37] Jafarpour S, Hirsch LJ, Gaínza-Lein M, Kellinghaus C, Detyniecki K. Seizure cluster: Definition, prevalence, consequences, and management. Seizure. 2019;68:9–15. 10.1016/j.seizure.2018.05.013 29871784

[38] Haut SR. Seizure clusters: Characteristics and treatment. Curr Opin Neurol. 2015;28(2):143–150. 10.1097/WCO.0000000000000177 25695133

[39] Epilepsy Foundation. Seizure Diary App - Nile [homepage on the Internet]. [cited 2022 Mar 9]. Available from: https://www.epilepsy.com/living-epilepsy/new-seizure-diary-app-nile

[40] Burman ME. Health diaries in nursing research and practice. Image J Nurs Sch. 1995;27(2):147–152. 10.1111/j.1547-5069.1995.tb00839.x 7622168

[41] Aitken LM, Rattray J, Hull A, Kenardy JA, Le Brocque R, Ullman AJ. The use of diaries in psychological recovery from intensive care. Crit Care. 2013;17(6):1–8. 10.1186/cc13164 PMC4056894 24351578

[42] Strandberg S, Vesterlund L, Engström Å. The contents of a patient diary and its significance for persons cared for in an ICU: A qualitative study. Intens Crit Care Nurs. 2018;45:31–36. 10.1016/j.iccn.2017.12.004 29295760

[43] Epilepsy Foundation. Finding the best dosage [homepage on the Internet]. 2020 [cited 2022 Mar 8]. Available from: https://www.epilepsy.com/learn/treating-seizures-and-epilepsy/seizure-and-epilepsy-medicines/finding-best-dosage

[44] Boggs JG. Seizure management in the intensive care unit. Curr Treat Options Neurol. 2021;23(11):1–15. 10.1007/s11940-021-00692-2 PMC8528936 34697528

[45] Palleria C, Di Paolo A, Giofrè C, et al. Pharmacokinetic drug-drug interaction and their implication in clinical management. J Res Med Sci. 2013;18(7):601–610. 24516494 PMC3897029

[46] Tatum WO IV, Winters L, Gieron M, et al. Outpatient seizure identification: Results of 502 patients using computer-assisted ambulatory EEG. J Clin Neurophysiol. 2001;18(1):14–19. 10.1097/00004691-200101000-00004 11290934

[47] Kerling F, Mueller S, Pauli E, Stefan H. When do patients forget their seizures? An electroclinical study. Epilepsy Behav. 2006;9(2):281–285. 10.1016/j.yebeh.2006.05.010 16824803

[48] Collier P. Poverty reduction in Africa. Proc Natl Acad Sci U S A. 2007;104(43):16763–16768. 10.1073/pnas.0611702104 17942702 PMC2040421

[49] Oye N, Salleh M, Iahad N. Challenges of e-learning in Nigerian university education based on the experience of developed countries. Int J Manag Inf Technol. 2011;3(2):39–48. 10.5121/ijmit.2011.3204

[50] World Bank. Access to electricity (% of population) – Sub-Saharan Africa [homepage on the Internet]. 2020 [cited 2022 Jan 10]. Available from: https://data.worldbank.org/indicator/EG.ELC.ACCS.ZS?locations=ZG&name_desc=true

